# Scaling and Clustering in Southern California Earthquake Sequences: Insights from Percolation Theory

**DOI:** 10.3390/e27040347

**Published:** 2025-03-27

**Authors:** Zaibo Zhao, Yaoxi Li, Yongwen Zhang

**Affiliations:** Data Science Research Center, Faculty of Science, Kunming University of Science and Technology, Kunming 650500, China; zaibo.zhao@stu.kust.edu.cn (Z.Z.); 20222111126@stu.kust.edu.cn (Y.L.)

**Keywords:** earthquakes, complex networks, percolation, phase transions

## Abstract

Earthquake activity poses significant risks to both human survival and economic development. However, earthquake forecasting remains a challenge due to the complex, poorly understood interactions that drive seismic events. In this study, we construct an earthquake percolation model to examine the relationships between earthquakes and the underlying patterns and processes in Southern California. Our results demonstrate that the model can capture the spatiotemporal and magnitude characteristics of seismic activity. Through clustering analysis, we identify two distinct regimes: a continuous increase driven by earthquake clustering, and a discontinuous increase resulting from the merging of clusters dominated by large, distinct mega-earthquakes. Notably, in the continuous increase regime, we observe that clusters exhibit a broader spatiotemporal distribution, suggesting long-range and long-term correlations. Additionally, by varying the magnitude threshold, we explore the scaling behavior of earthquake percolation. The robustness of our findings is confirmed through comparison with multiple shuffling tests.

## 1. Introduction

Earthquakes are among the most destructive natural disasters, marked by complex spatiotemporal dynamics. Previous studies have identified several empirical laws that govern seismic activity, including the Gutenberg-Richter law, which describes the distribution of earthquake energies follows a power law [1], and the Omori-Utsu law, which characterizes the power-law decay of aftershock frequency over time [2,3]. Additionally, scaling and power-law relationships have been established for the waiting times between earthquakes [4,5,6,7,8]. While earthquake magnitudes were considered independent, with their frequency-magnitude distribution following the Gutenberg-Richter law, some studies suggest that consecutive earthquake magnitudes may exhibit correlations [6,9,10,11,12,13].

Long-range correlations and long-term memory effects in seismic activity have also been observed [7,8,14,15,16], with large-magnitude mainshocks influencing subsequent earthquake sequences over extended timescales [17]. A key feature of earthquake activity is clustering, which plays a crucial role in seismological research, including aftershock identification, earthquake simulation, and forecasting. Clustering occurs along plate boundaries, major faults, and in the form of aftershocks following large earthquakes, often involving foreshocks as well [18,19,20,21,22]. This behavior reflects underlying triggering mechanisms, such as stress transfer, which induces aftershocks near a mainshock [23,24,25], or even triggers remote seismic events through dynamical processes [26,27,28]. Understanding these clustering patterns is critical, as they offer insights into the spatiotemporal evolution of seismic activity and its potential for long-term forecasting.

To explore these complex patterns, network theory has become an essential tool in the analysis of complex systems, significantly advancing earthquake research. Earthquake complex networks enable the analysis of seismic characteristics [29], the classification of events into foreshocks, mainshocks, and aftershocks [30,31], and the mapping of seismic data onto scale-free and small-world networks. These networks can exhibit assortative mixing properties, which are crucial for mainshock prediction through the identification of nodes with high betweenness centrality [32]. Additionally, percolation theory, a concept derived from network theory and statistical physics, has been successfully applied in various complex system studies. Percolation models have provided insights into processes such as heat and nutrient transport in sea ice [33], the interplay between water dynamics and long-term topographical evolution, and the characterization of lunar surface features [34]. Furthermore, these models have deepened our understanding of Earth’s geometric phase transitions, identified key nodes in Earth’s evolutionary processes [35], and been applied to predict the El Niño phenomenon [36,37]. The application of percolation theory to earthquake clustering thus represents a promising approach for uncovering the underlying processes that govern complex seismic activity.Many natural phenomena also exhibit phase transition-like behavior [38,39].

In this study, we use percolation theory to investigate the complex relationships underlying earthquake clustering. We analyze seismic activity in Southern California to examine the driving mechanisms behind earthquake cluster formation and expansion. By studying the scaling behavior of earthquake percolation, we explore the phase transition of earthquake clustering and compare our results with several shuffling models to identify non-trivial physical features.

## 2. Materials and Methods

### 2.1. Data

**Figure 1:**
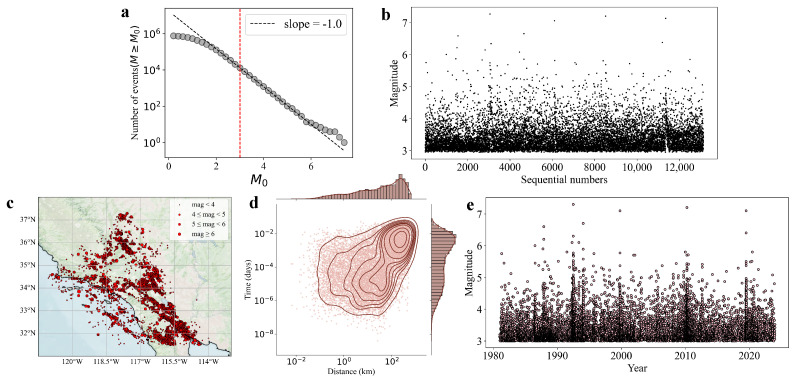
Basic spatiotemporal characteristics of earthquakes. (**a**) Cumulative distribution of earthquake magnitudes. (**b**) Dithered magnitudes versus sequential numbers of the earthquake events in the study region. The timescale is equalized for observed earthquakes. The magnitudes are dithered with random errors uniformly in [−0.05, 0.05]. (**c**) Spatial distribution of earthquakes in Southern California. (**d**) Scatter plot (log−log) of time intervals and distance intervals between two neighboring earthquakes in time. (**e**) Magnitude distribution with earthquake occurrence time.

The dataset used in this study is the Southern California Earthquake Catalogue, covering the period from 1981 to 2023. It is available for download from the Southern California Earthquake Data Center (SCEDC) at https://scedc.caltech.edu/data/alt-2011-dd-hauksson-yang-shearer.html (accessed on 8 December 2024) [40]. In earthquake catalog data, the earthquake could be missing due to the weak detection of the seismic network. From Figure 1a, the count of earthquakes with the condition as a function of magnitude threshold, it can be observed that for, the data generally conform to the Gutenberg-Richter law, except for minor deviations in higher-magnitude earthquakes due to limited data availability. Therefore, we define the magnitude threshold as $M_{c}=3$, meaning that only earthquakes with magnitude $M\geq M_{c}=3$ are considered. However, it is important to note that even for $M\geq M_{c}=3$, due to the influence of high-magnitude earthquakes, a small proportion of missing earthquake events can still be observed in the catalog, as indicated by the dithered magnitudes versus sequential numbers (Figure 1b) [41].

Figure 1c–e illustrates the spatial distribution of selected seismic events, along with the distribution of time intervals and geographic distances between earthquakes with adjacent occurrence times. The data reveal a clear spatial clustering of earthquakes, predominantly occurring along faults and plate boundaries, as shown in Figure 1c. Figure 1d indicates that most earthquakes are characterized by both short time intervals and short distances, with a noticeable correlation between the two. Additionally, Figure 1e highlights several earthquake clusters in times that appear to coincide with the occurrence of large seismic events in Southern California.

### 2.2. Earthquake Complex Network and Percolation Model

We construct an earthquake complex network by representing each seismic event as a node. The connection strength for nodes *i* to *j* is quantified by the edge distance $\eta_{ij}$, which incorporates the time, space, and magnitude information of the events, each earthquake is connected to events that occur later in time. Specifically, $\eta_{ij}$ is defined as follows [31,42]:

(1) $$\eta_{ij}=t_{ij}{\left(r_{ij}\right)}^{d_{f}}10^{-bm_{i}},0\leq t_{ij}<1.$$

The time interval between events *i* and *j* is given by $t_{ij}=t_{i}-t_{j}$ (in years), with only $t_{ij}$ values smaller than one year considered. The spatial distance $r_{ij}$ (in kilometers) between events *i* and *j* is used, with the depth of the earthquakes ignored in favor of surface distance. The variable $m_{i}$ denotes the magnitude of event *i*, and $d_{f}$ is the fractal dimension of the spatial distribution of earthquakes. In this study, we set $d_{f}=1.6$ and b=1 associated with the Gutenberg-Richter law, as suggested in previous works [31,42]. A smaller value of $\eta_{ij}$ corresponds to a stronger connection for events *i* to *j*.

After constructing the earthquake complex network, we implement a percolation model. First, all edge distances η in the network are sorted in ascending order. We then sequentially add edges, starting with the edge corresponding to the strongest connection (the smallest η), and continue with the next strongest, and so on. According to percolation theory, a cluster is defined as a set of nodes connected by at least one edge [43,44]. The relative size of the largest cluster, *s*, is used as “the order parameter”—a physical quantity that quantifies the macroscopic state of the system and serves as a key indicator of phase transitions and critical behavior [45]. In the context of the earthquake complex network, the size of a cluster is defined by the number of seismic events it contains, correspondingly, the relative size of a cluster is the cluster size divided by the total number of considered seismic events.

To assess changes in cluster size, we calculate the gap in the relative size of the largest cluster, Δs, after each edge is added [46,47]:

(2) $$\Delta s_{i}=s_{i+1}-s_{i}\;,$$

where $s_{i}$ is the relative size of the largest cluster after the addition of the *i*-th edge. By analyzing the maximum gap in the relative size of the largest cluster, $\Delta s_{\max}$, we identify potential discontinuities in the order parameter *s*.

## 3. Results

### 3.1. Percolation Process in Earthquake Complex Network

**Figure 2:**
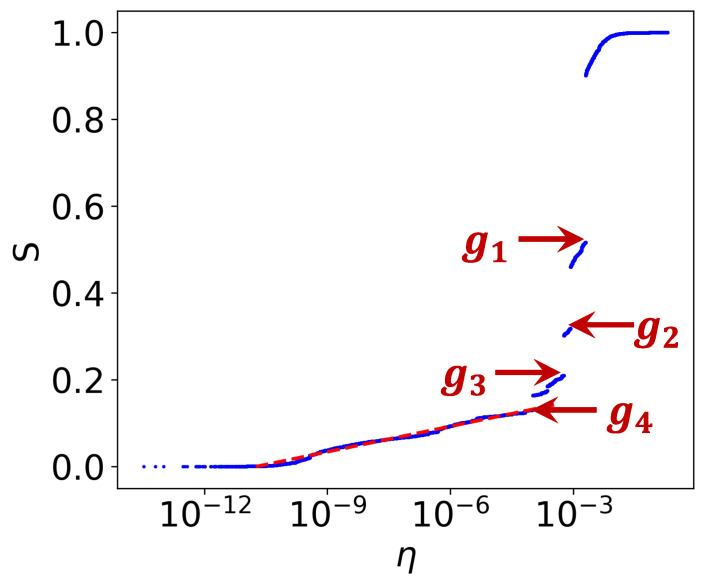
Percolation on the real earthquake complex network. The relative size of the largest cluster *s* as a function of the edge distance η (with η presented on a logarithmic scale). $g_{1}$ to $g_{4}$ represent the first to fourth largest gaps in the order parameter. The red line represents the linear fit of the continuous increasing trend before the occurrence of $g_{4}$.

To quantify the connection strength between earthquakes, we use Equation (1) to calculate the edge distance and construct earthquake complex networks. The percolation model is then applied to these networks (for details, see Methods). Figure 2 shows the variation in the relative size of the largest cluster (the order parameter, *s*) in the earthquake network percolation model as a function of the edge distance. A clear and abrupt phase transition is observed around $\eta_{ij}=10^{-3}$ (Figure 2).

The change in the order parameter follows two distinct regimes. Initially, *s* increases logarithmic according to the relationship $s\approx 0.0196\log_{10}\eta +0.21$, reflecting the dominance of large magnitude earthquakes that preferentially form strongly correlated earthquake clusters. During this phase, multiple major earthquakes independently generate their own clusters. Subsequently, the growth of *s* shifts from a steady continuous increase to a sudden jump. To understand the driving mechanism behind this phase transition, we analyze the clustering structure of the earthquake complex network before the four largest gaps in the order parameter, Δs, as identified in Figure 2 ($g_{1}$–$g_{4}$).

**Figure 3:**
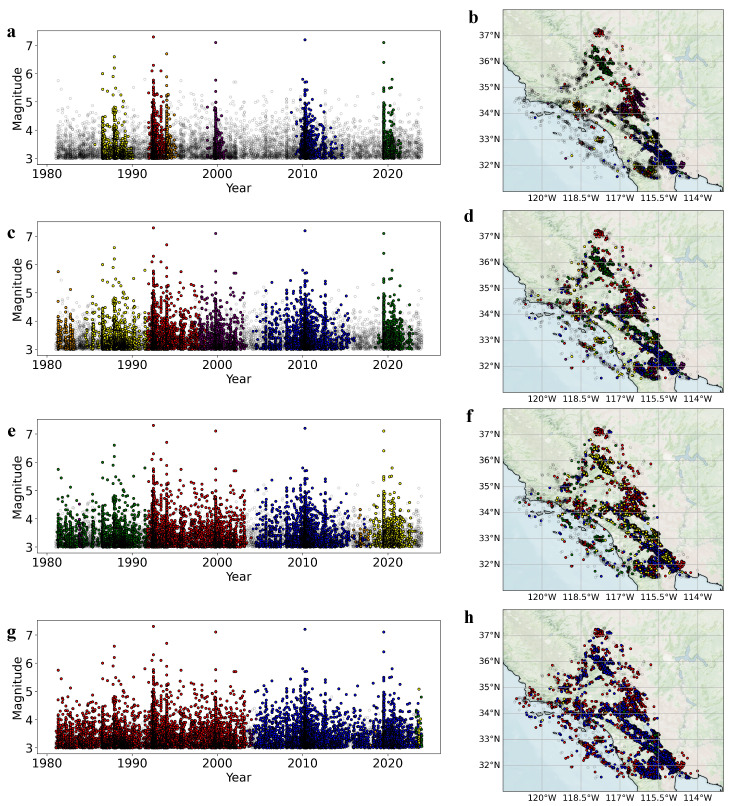
Clusters in the earthquake complex network before the occurrence of gaps. (**a**) Magnitudetime (M-T) plot of clusters before the occurrence of the fourth-largest gap, $g_{4}$, plotted by time and latitude. Each point represents an earthquake, with red, blue, green, yellow, purple, and orange corresponding to earthquakes in the first to sixth largest clusters. Semi-transparent hollow points indicate other earthquakes. (**b**) Same as (**a**), but showing the spatial distribution. (**c**–**h**) show the same as (**a**,**b**) for the gaps $g_{3}$, $g_{2}$, and $g_{1}$, respectively.

Figure 3a,b present the six largest clusters in the network before the occurrence of the fourth-largest gap, $g_{4}$. Comparing this with the earthquake occurrence times and magnitudes in Figure 1c, it is evident that before the jump in cluster size, clusters dominated by large-magnitude earthquakes form first, which is consistent with aftershock triggering. The gap $g_{4}$ arises from the merging of two distinct mainshock clusters (the red and orange clusters in Figure 3a,b) as the edge distance η increases, causing the growth pattern of the largest cluster to shift from a continuous to an abrupt increase. As η continues to increase, preferentially formed clusters merge further (e.g., the red and purple clusters in Figure 3c,d, and the red and green clusters in Figure 3e,f). Eventually, two dominant clusters emerge in the network (the red and blue clusters in Figure 3g,h), each occupying nearly fifty percent of the network. Their subsequent merging causes the largest jump in cluster size ($g_{1}$), after which all nodes in the network rapidly connect, and the relative size of the largest cluster reaches 1.

It is noteworthy that before and after the phase transition, the underlying mechanisms driving the evolution of clusters are not identical. When *s* exhibits a continuously increasing trend, aside from the mainshock–aftershock pairs that are proximate in both time and space, the earthquake events within the clusters in the model establish longer-range connections in time and space through direct or indirect interactions. Specifically, within the clusters there exist earthquake events that are temporally close yet spatially extensive, as exemplified by the red and purple clusters in Figure 3a, whose spatial distributions essentially span the entire latitudinal range of the region, the two most distant earthquake events in red cluster span a distance exceeding 500 km; as shown in Figure 3b, the earthquake events in the clusters are not concentrated in any single area but are widely distributed throughout the entire Southern California seismic belt. At the same time, there also exist events that are spatially proximate yet exhibit long temporal spans, such as the yellow and blue clusters in Figure 3a, with the temporal scale extending over five years. From the statistical perspective, both “temporally close yet spatially extensive” and “spatially proximate yet exhibiting long temporal spans” can indicate strong correlations between earthquakes, though the underlying mechanisms may differ entirely. This demonstrates that earthquake activity exhibits both long-range and long-term correlations, which may be attributed to remote triggering and the influence of long-term memory effects. When *s* transitions to a jump-like increasing trend, clusters dominated by large-magnitude earthquakes begin to merge, and the differing association distances between clusters lead to a sequential merging process; for instance, as shown in Figure 3c, the red cluster first merges with the purple cluster, resulting in the jump $g_{3}$, after which the orange and yellow clusters merge, forming, respectively, the green and red clusters depicted in Figure 3e, whose subsequent combination gives rise to the jump $g_{2}$. These phase transitions may reflect the shift from earthquake aftershock clustering to the uncorrelated seismicity.

### 3.2. Validation Using the Shuffled Model

We next assess the significance and stability of the abrupt transitions observed in the earthquake complex network’s percolation model by constructing shuffled models. These models disrupt the spatiotemporal characteristics of the earthquake catalog. Specifically, for the geographic locations of earthquake occurrences, we employ a method that randomly generates locations within the latitude range of 30° N to 38° N and the longitude range of 112° W to 122° W. To simulate both completely random geographical distributions and those that preserve the real fractal characteristics, we first compute the probability distribution of actual earthquakes within a 0.01° grid and then generate random locations within the same grid accordingly. For the temporal intervals between earthquakes, we calculate the real earthquake occurrence rate, $\mu_{0}\approx 0.836$, and generate synthetic inter-event intervals using an exponential distribution with rate parameter $\mu_{0}$. Earthquake magnitudes are randomly assigned from the real catalog.

By combining these approaches, we construct five distinct shuffled models as follows:

- Shuffled Model 1: Completely random locations; simulated inter-event intervals; randomly assigned magnitudes.Construct a fully randomized model that preserves none of the original sequence’s characteristics.
- Shuffled Model 2: Locations based on probability distribution; simulated inter-event intervals; randomly assigned magnitudes.Construct a only keep the fractal characteristics of the original sequence model.
- Shuffled Model 3: Locations based on probability distribution; real earthquake inter-event intervals; randomly assigned magnitudes.Construct a model that preserves both the fractal characteristics and the event time intervals of the original sequence.
- Shuffled Model 4: Real earthquake locations; simulated inter-event intervals; randomly assigned magnitudes.Construct a model that strictly preserves the earthquake events locations.
- Shuffled Model 5: Real earthquake locations; real earthquake inter-event intervals; randomly assigned magnitudes.Construct a model that strictly preserves both the earthquake events locations and events time intervals.

**Figure 4:**
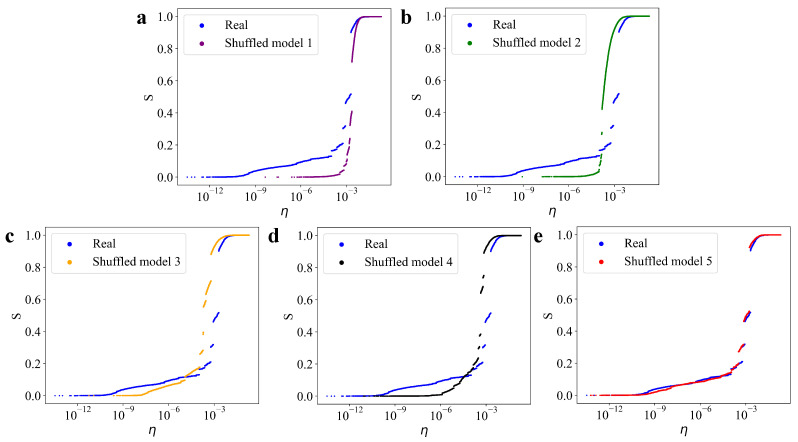
Percolation model comparison between the real data and the shuffled model. Comparison of *s* variations between the real data and the shuffled models for (**a**) shuffled model 1, (**b**) shuffled model 2, (**c**) shuffled model 3, (**d**) shuffled model 4, (**e**) shuffled model 5.

Figure 4 compares the results from the shuffled models to those of the percolation model constructed from the real earthquake catalog. It is evident that only Shuffled Model 5 exhibits characteristics similar to those of the real earthquake catalog, both in the initial linear growth phase and the subsequent discontinuous jumps. When either the temporal or spatial features are disrupted, the resulting patterns deviate from those observed in the real data. Shuffled models 1–4 lack the full spatiotemporal characteristics of real earthquakes, meaning that the network nodes are randomly connected. This explains why the order parameters of models 1–4 exhibit an instantaneous growth pattern, akin to traditional percolation models.

**Figure 5:**
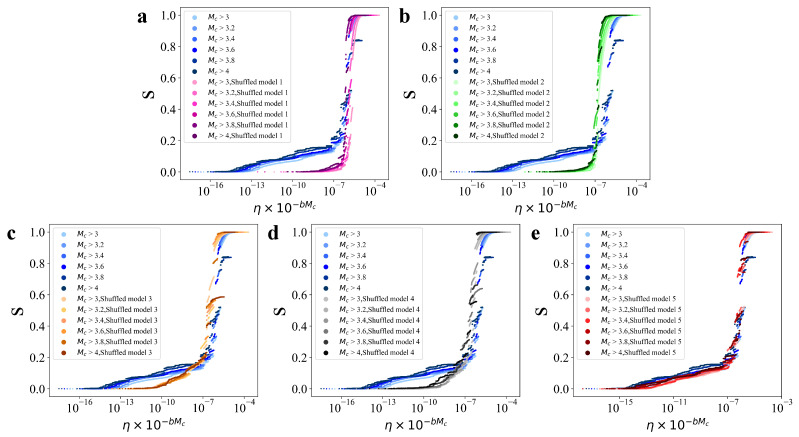
Earthquake percolation model with different magnitude thresholds for real data and shuffled models. The relative size of the largest cluster, *s*, as a function of the rescaled edge strength, $\eta \times 10^{-bM_{c}}$, for (**a**) shuffled model 1, (**b**) shuffled model 2, (**c**) shuffled model 3, (**d**) shuffled model 4, and (**e**) shuffled model 5.

To further assess the robustness of the phase transition observed in both the real data and shuffled models, we investigate the effect of varying the selected magnitude threshold, $M_{c}$, in the earthquake catalog. Specifically, we consider five different thresholds: $M_{c}=3.0,3.2,3.4,3.6,3.8,4.0$, and perform percolation model calculations for each case. The results, shown in Figure 5, reveal that after rescaling η by a factor of $\eta \times 10^{-bM_{c}}$, which is consistent with Gutenberg-Richter’s law, the data across all scales converge to a consistent trend. This indicates that our findings are independent of the chosen magnitude threshold, and the model can capture the unified spatiotemporal-magnitude characteristics of earthquakes at any selected threshold.

### 3.3. Examination of Finite-Size Effects

**Figure 6:**
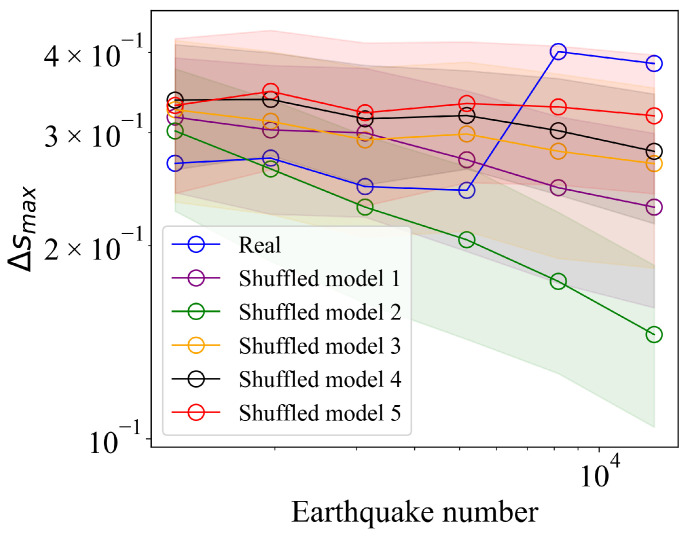
Finite-size effects in the earthquake complex network percolation model. Log-log plot of the largest gap, $\Delta s_{\max}$, versus the number of earthquakes for real data and shuffled models. For the shuffled models, circles represent the mean values obtained from 1000 computations, and the shaded regions indicate the standard deviation as error bars.

We next examine the finite-size effects on the percolation process within the earthquake complex network. According to the Gutenberg-Richter law, which elucidates the relationship between earthquake frequency and magnitude, altering $M_{c}$ enables us to change the number of earthquake events in the network, thereby adjusting the system size. Consistent with the previous approach, we conduct analyses using $M_{c}=3$, 3.2, 3.4, 3.6, 3.8, and 4.0. Specifically, we analyze the variation of $\Delta s_{\max}$ (the largest gap) as a function of system size. In percolation models, if a system undergoes a continuous phase transition, $\Delta s_{\max}$ should approach zero as the system size increases [36,48]. To test this, we conducted 1000 random shuffling experiments for each shuffled model, with the results shown in Figure 6. The system size is represented by the number of earthquake events in each model. For shuffled models 1–4, which do not fully preserve the spatiotemporal characteristics of real earthquakes, we observe a decreasing trend in $\Delta s_{\max}$ as the system size increases. This trend is most pronounced in shuffled model 1, which completely lacks real earthquake spatiotemporal features, followed by shuffled model 2, which retains only the fractal characteristics of earthquake locations. Models 3 and 4, which retain either the temporal or spatial characteristics of real earthquakes, show a weaker decreasing trend. In these models, the lack of complete spatiotemporal characteristics results in a lack of a stable gap, undermining the characteristic cluster behavior.

In contrast, shuffled model 5, which fully preserves the spatiotemporal characteristics of real earthquakes, shows a more stable $\Delta s_{\max}$ as the system size increases, approaching zero. This suggests that retaining spatiotemporal features enhances the abrupt transitions observed in the earthquake complex network percolation model. However, the percolation model constructed from real earthquake data exhibits a notable increasing trend in $\Delta s_{\max}$ as the system size grows. This trend is rare in percolation models based on natural networks and is not reproduced by shuffled model 5. This suggests that the phenomenon observed in our model is not merely a result of spatiotemporal scales but emerges from the complex interplay of temporal, spatial, and magnitude characteristics. Furthermore, it is important to note that, in addition to the correlation between earthquake magnitudes, this characteristic may also be influenced by the incomplete detection of low-magnitude events in the earthquake catalog. This could lead to an underestimation of the clustering effect of weak earthquakes, resulting in an overall increasing trend in the gap. However, overall, the real data do not exhibit the decreasing trend observed in shuffled models 1–4, which is sufficient to demonstrate that the gap in our model remains stable and significant.

## 4. Conclusions

This study reveals two clustering evolution patterns in the Southern California seismic sequence by constructing an earthquake complex network percolation model: during the continuous growth phase, remote stress transfer triggered by major earthquakes and long-term memory effects result in the clustering of seismic swarms dominated by large magnitudes over large spatial and temporal scales, At this stage, the farthest distance between earthquake events within a cluster can reach 500 km, and the longest time span of a cluster can extend beyond five years; however, once η reaches a critical value, the clusters dominated by major earthquakes in the system rapidly merge, leading to a pronounced discontinuous jump in the maximum cluster size and signifying an abrupt change in the overall system structure. Quantitative analysis of the maximum jump, $\Delta s_{\max}$, indicates that this phase transition behavior is not a mere statistical coincidence, but rather a reflection of the intrinsic physical mechanisms underlying the earthquake occurrence process.

In order to further validate the authenticity of this phenomenon, we constructed several shuffled models that selectively disrupt or preserve the spatial, temporal, and magnitude characteristics of the real data. The results show that only in the shuffled model that fully preserves the true spatiotemporal characteristics (shuffled model 5) can the continuous growth and discontinuous jump phenomena observed in the real data be partially reproduced; whereas when any key characteristic is disrupted, the system exhibits an instantaneous growth behavior similar to that of the traditional percolation model. This comparative result underscores the critical role of spatiotemporal coupling in the formation of complex clustering behavior in real seismic sequences. Moreover, by varying $M_{c}$ and scaling η, we found that the data exhibit a uniform growth trend across different thresholds. This result not only demonstrates the universality of the spatiotemporal–magnitude coupling captured by the model, but also further indicates that the intrinsic complex dynamics of the Southern California seismic sequence are a key driving force behind its clustering phenomena.

Our study provides a statistical physics perspective for characterizing the nonlinear and phase transition processes in seismic activity, and it could also contribute to improving earthquake forecasting models. Furthermore, the specific mechanisms of long-range triggering in earthquake clustering, as well as whether long-term memory and the critical value of η are affected by the seismic background or other factors, along with the clustering evolution mechanisms at different regions and scales, warrant further investigation.

## Data Availability

The data used in this study are all open-access. The data can be downloaded from SCEDC (Southern California Earthquake Data Center) at https://scedc.caltech.edu/data/alt-2011-dd-hauksson-yang-shearer.html (accessed on 8 December 2024).
